# Higher protein intake is associated with a lower likelihood of frailty among older women, Kuopio OSTPRE-Fracture Prevention Study

**DOI:** 10.1007/s00394-019-01978-7

**Published:** 2019-05-07

**Authors:** Masoud Isanejad, Joonas Sirola, Toni Rikkonen, Jaakko Mursu, Heikki Kröger, Sarang Latif Qazi, Marjo Tuppurainen, Arja T. Erkkilä

**Affiliations:** 1 Institute of Public Health and Clinical Nutrition, University of Eastern Finland, Yliopistonranta 1C, PO Box 1627, 70211 Kuopio, Finland; 2 Institute of Ageing and Chronic Disease, 23 University of Liverpool, Liverpool, L69 3GA UK; 3 Kuopio Musculoskeletal Research Unit, University of Eastern Finland, Kuopio, Finland; 4 Department of Orthopaedics and Traumatology, Kuopio University Hospital, Kuopio, Finland; 5 Department of Obstetrics and Gynaegology, Kuopio University Hospital, Kuopio, Finland

**Keywords:** Protein intake, Animal protein, Plant protein, Frailty, Older women

## Abstract

**Purpose:**

Nordic nutrition recommendations (2012) suggest protein intake ≥ 1.1 g/kg body weight (BW) to preserve physical function in Nordic older adults. However, no published study has used this cut-off to evaluate the association between protein intake and frailty. This study examined associations between protein intake, and sources of protein intake, with frailty status at the 3-year follow-up.

**Methods:**

Participants were 440 women aged 65─72 years enrolled in the Osteoporosis Risk Factor and Prevention–Fracture Prevention Study. Protein intake g/kg BW and g/d was calculated using a 3-day food record at baseline 2003─4. At the 3-year follow-up (2006─7), frailty phenotype was defined as the presence of three or more, and prefrailty as the presence of one or two, of the Fried criteria: low grip strength adjusted for body mass index, low walking speed, low physical activity, exhaustion was defined using a low life-satisfaction score, and weight loss > 5% of BW. The association between protein intake, animal protein and plant protein, and frailty status was examined by multinomial regression analysis adjusting for demographics, chronic conditions, and total energy intake.

**Results:**

At the 3-year follow-up, 36 women were frail and 206 women were prefrail. Higher protein intake ≥ 1.1 g/kg BW was associated with a lower likelihood of prefrailty (OR = 0.45 and 95% confidence interval (CI) = 0.01–0.73) and frailty (OR = 0.09 and CI = 0.01–0.75) when compared to protein intake < 1.1 g/kg BW at the 3-year follow-up. Women in the higher tertile of animal protein intake, but not plant protein, had a lower prevalence of frailty (*P* for trend = 0.04).

**Conclusions:**

Protein intake ≥ 1.1 g/kg BW and higher intake of animal protein may be beneficial to prevent the onset of frailty in older women.

## Introduction

Frailty can be defined as a state of augmented sensitivity and vulnerability to external stressors in old age, and poor resolution of homeostasis after a stressor event, which increases the risk of adverse health outcomes and disability [1]. The Fried frailty phenotype classifies frailty as the presence of three or more of the following five components: weakness, slowness, low physical activity, exhaustion, and weight loss [2], and prefrailty as the presence of one or two of the Fried phenotype criteria. In a recent systematic review carried out in 61,500 community-dwelling individuals aged 65 and older, the overall prevalence of frailty was estimated to be 10.7%, and 41.6% were prefrail with one or two components of Fried frailty phenotype [3]. However, because of the varied definitions of frailty status used in those studies, the reported prevalence differed substantially, ranging between 4.0 and 59.1%.

Decline in physical function and muscle strength is the major risk factor for frailty [4] and it has been shown that protein intake is associated with better physical function and muscle strength in older people [5]. In a randomized controlled trial by Tieland et al. (2012), 24 weeks of dietary protein supplementation (15 g protein at breakfast and lunch) improved physical performance, but did not increase skeletal muscle mass in frail elderly people [6]. However, only a few studies showed an association between protein intake and a risk of frailty [7, 8]. Recent results have been controversial; Shikany et al. reported that quintiles of protein intake (% of energy) were not associated with a risk of frailty [9].

There is growing support for the concept that greater protein intake may preserve physical function in older adults [10, 11]. The anabolic response to amino acid intake may be blunted in older people, particularly if they have low intakes of protein [12]. Moore et al. claimed that the relative amount of protein required to maximize muscle protein synthesis was greater in older men as compared with their younger counterparts [13]. However, most of the previous studies assessed protein intake continuously or using population-based cut-offs [7, 9] but not according to available dietary guidelines for the older people. Only in one recent study, among 1345 French community-dwelling older subjects, was protein intake ≥ 1 g/kg body weight (BW) associated with a lower prevalence of frailty [8]. Nordic nutrition recommendations 2012 (NNR) in particular suggested dietary protein intake of at least 1.1–1.3 g protein/kg BW in older adults [14].

In addition, animal protein intake may be associated with muscle strength in older adults [16], which may be associated with a lower risk of frailty; whereas, plant-based protein sources may have limited potential to stimulate the skeletal muscle anabolic response. The exact reasons are not understood but it might be that plant proteins are considered to have a lower content of essential amino acids compared to animal protein sources [15, 16]. Nevertheless, knowledge regarding the association between adequate protein intakes, according to recommendations, and sources of protein intake with frailty are limited. We hypothesized that the prevalence of frailty and prefrailty was lower among older women consuming ≥ 1.1 protein/kg BW compared to those with lower intakes. In addition, we examined the association between sources of protein intake, i.e., animal protein and plant protein, with frailty and prefrailty.

## Materials and methods

### Study population

Data for the present study were collected from the Osteoporosis Risk Factor and Prevention–Fracture Prevention Study (OSTPRE-FPS), which began in 2003 in Kuopio, Finland [17]. The OSTPRE-FPS was a randomized population-based open trial with a 3-year follow-up in 3432 women (aged 66–71 years). The primary aim of the study was to determine whether vitamin D and calcium supplementation would be effective in preventing falls and fractures in postmenopausal women. The intervention and control groups had 1718 and 1714 subjects, respectively [17, 18].

OSTPRE-FPS study investigated the effect of vitamin D and calcium supplementation on fracture prevention in a sample of 750 women (375 from each of the intervention and control groups) which was randomly selected from the 3432 women and underwent detailed examination at baseline and follow-up, including measurements of body composition, clinical, physical function, and laboratory tests. Between randomization and the actual start of the intervention, 237 (31.6%) subjects withdrew, and ten (1.3%) were lost during the follow-up. At the end of the trial (*n* = 593, 79.0%), 306 (40.8%) and 287 (38.2%) subjects in the intervention and control groups of the subsample, respectively, completed the follow-up measurements. Out of those, 554 (93%) women returned a valid food record at the baseline, and 39 women did not return the food record, or it was incomplete. Data for the four-item life-satisfaction scale used as a surrogate of exhaustion were missing for 114 (19.2%) of these women. Thus, for this study, the final analytical data included 440 (58% of 750) women, who had all the components for the calculation of the Fried frailty phenotype available at the 3-year follow-up. The baseline characteristics were similar between women who were included or not included in this analysis.

The power analysis was performed based on the incidence of fractures [18]. There was no a priori power analysis to calculate the size of the subsample of 750 women randomly selected from the 3432 women at the baseline. All participants provided written permission for participation. The ethical committee of Kuopio University Hospital approved the study in October 2001. The study was registered at Clinical trials.gov by the identification NCT00592917.

### Questionnaires and measurements

All clinical measurements were performed at Kuopio Musculoskeletal Research Unit of the Clinical research center of the University of Kuopio. The OSTPRE-FPS baseline questionnaire contained questions on income per month (euros), age at menopause (years), chronic diseases, and years of hormone therapy. The physical function assessments have previously been explained in detail [19]. Grip strength (kg) was measured (JAMARTM handgrip dynamometer; Sammons Preston, Bolingbrook, IL) from the nondominant hand while sitting on a bench, with the forearm flexed from the elbow at a 90° angle, near the torso. A total of three attempts were recorded, with approximately 30 s of resting time between the tests. Close attention was paid to make all three attempts in a similar, fixed posture. The best attempt out of the three was recorded as the maximal result. The intraclass correlation coefficient for grip strength measurements was 0.93 [20]. Walking speed was assessed by asking women to walk the 10-m distance at their usual pace. Time was recorded and the walking speed was calculated as m/s.

### Frailty ascertainment

Frailty was assessed after 3 years of follow-up, using criteria developed by Fried and colleagues or their surrogates [2] explained in Supplementary Table 1. Fried et al. used a cut-off for grip strength stratified by gender and BMI, but due to our younger study population only five women had grip strength under this cut-off [2]. Thus, we categorized women into quartiles of grip strength adjusted for BMI, the lowest quartile < 0.67 kg/m^2^. Walking speed was first adjusted for height [2]; women who did not complete the test were considered in the same group as women in the lowest quartile of walking speed adjusted for height < 0.51 m/s. Type of physical activity (summer and winter) and its frequency (hours per week) were obtained by questionnaire at baseline and follow-up. Reported weekly physical activity was used to create a long-term physical activity variable by summing up the average weekly hours spent on physical activity.

**Table 1:** Baseline characteristics of the participants according to Nordic nutrition recommendation for protein intake cut-off (g/kg body weight)

	Protein intake				*P*
	< 1.1 g/kg body weight (*n* = 301)		≥ 1.1 g/kg body weight (*n* = 139)		
	Mean	SD	Mean	SD	
Age (year)	67.8	1.8	67.7	1.8	0.545
Current smoking *n* (%)	24 (6.4)		2 (1.2)		0.030
Hormone therapy use n (%)	158 (43.4)		91 (61.1)		0.002
BMI (kg/m^2^)	28.4 (4.4)		25.3 (2.9)		< 0.001
BMI category (kg/m^2^), *n* (%)					< 0.001
Normal, ≤ 24.9	59 (16.4)		54 (35.8)		
Overweight, 25─29.9	153 (42.6)		82 (54.3)		
Obese ≥ 30	147 (40.9)		15 (9.9)		
Income per month (euros)	850	299	850	299	0.896
Fried frailty components					
Grip strength (kg)	25.0	5.7	24.4	5.0	0.242
Grip strength adjusted for BMI	0.94	0.26	0.99	0.20	0.019
Walking speed (m/s)	1.51	0.36	1.65	0.32	0.007
Belong to lowest quartile of walking speed adjusted for height (m/s)	92 (27.3)		25 (16.3)		0.005
Physical activity (hours/week)	12.9	8.2	14.4	8.4	0.050
Belong to lowest quartile of physical activity (hours/week) n (%)	99 (25.6)	31 (18.8)	0.052		
Belong to lowest quartile of grip strength adjusted for BMI *n* (%)	98 (27.8)		25 (16.1)		0.003
Exhausted n (%)^a^	53 (16.1)		13 (9.4)		0.038
Weight loss > 5% of body weight n (%)	41 (11.4)		12 (7.7)		0.134
Dietary factors					
Energy intake (kJ/d)	6027	1337	7790	1306	0.693
Protein (g/d)	61.8	10.2	72.5	11.8	< 0.001
Protein (g/kg body weight)	0.79	0.16	1.30	0.19	< 0.001
Total animal protein (g/kg body weight)	0.51	0.14	0.90	0.18	< 0.001
Total animal protein (g/d)	36.0	10.2	46.7	11.8	< 0.001
Animal protein sources					
Dairy protein (g/d)	19.3	8.6	29.8	9.6	0.001
Poultry and meat protein (g/d)	11.5	7.3	16.2	7.8	0.001
Fish protein (g/d)	5.8	3.8	11.0	4.7	0.001
Egg protein (g/d)	2.0	1.7	2.2	1.8	0.140
Total plant protein (g/kg body weight)	0.27	0.07	0.39	0.11	< 0.001
Total plant protein (g/d)	23.8	4.1	24.3	4.7	0.242
Plant protein sources					
Protein from cereal (g/d)	15.6	4.8	19.8	5.6	0.001
Other plant protein (g/d)^b^	3.1	1.7	4.1	2.5	0.001
Alcohol (portions/week)	0.7	1.2	0.9	1.5	0.118

Independent sample *t* test and Chi-square test were used to evaluate the differences between participants’ characteristics across protein intake categories (g/kg body weight)

*BW* body weight

^a^Life-satisfaction score 4–12

^b^Other plant protein sources included all vegetable sources, legumes, nuts, fruits and berries

Fried and colleagues used self-reported exhaustion to indicate poor endurance [2]. Self-reported exhaustion was defined by two questions from the Center for Epidemiologic Studies Depression Scale (CESD) (“I felt that anything I did was a big effort” and “I felt that I could not keep on doing things”) [31]. In these data, the measures of life satisfaction have been investigated previously and used as a surrogate for the frailty components [32]. A brief 4-item life-satisfaction scale which was used in this study was previously linked with depression in adults [33] and identified those with increased risk for several adverse health outcomes such as morbidity, mortality and suicide, as well as both psychiatric and somatic disability and morbidity [34, 35]. The items in the questionnaire include current feelings of (1) interest and (2) happiness in life, (3) ease of living; and (4) feelings of loneliness, total score range 4–20, higher score indicating lower life satisfaction. Further, life satisfaction was used as a three-category variable (the satisfied, LS = 4–6; the intermediate group, LS = 7–11; the dissatisfied, LS = 12–20) (the satisfied, LS 4–11; the dissatisfied, LS = 12–20) [32]. Dissatisfied group was considered as a surrogate for exhaustion in frailty ascertainment.

According to the Fried recommendation, we used weight loss over 5% of body weight in the previous 3 years as a cut-off because the intentionality of the weight loss was not questioned in this study. It is noteworthy that using a weight loss indicator limited this study to only define frailty at the 3-year follow-up. To produce the final frailty score if the subject belonged the lowers quartile of grip strength adjusted for BMI, walking speed adjusted for height, and physical activity they received score of one point, otherwise 0 score was assigned. Those with a total life-satisfaction points ranging 12–20 received a score = 1, and women who lost body weight ≥ 5% received a score = 1. To define frailty, each participant received a frailty score in the range of 0–5. Women with a score of three or more were considered as frail, a score of 1–2 as prefrail and those with a score of 0 as a referent group (not frail).

### Dietary intakes

Dietary intake was assessed using a 3-day food record at baseline. A questionnaire and the instructions were sent to participants beforehand, and they were returned at the baseline visit. Participants were instructed to write down their food and beverage intake, along with the amount consumed using household measures for three consecutive days, with 2 days during the week and 1 day at the weekend (Saturday or Sunday) [21]. The types of fats used on bread, in cooking and in baking were recorded. In the case of uncertainties about food record, a nutritionist called the participant for more clarification. Nutrient intakes were calculated using Nutrica dietary analysis software (version 2.5, Finnish Social Insurance Institute, Turku, Finland) based on the national database of the Finnish Social Insurance Institution. Assessment of underreporting has previously been described and none of the participants was excluded due to low energy intake [22]. The collected data provided estimated intakes of animal protein (dairy products, eggs, fish, poultry and meat) and plant protein (cereals, legumes, vegetables, fruit and berries) in addition to total protein intake.

## Potential confounders

Potential confounders were selected a priori based on their reported association with either diet or frailty in the literature. Data on demographic characteristics (age, income per month), medical history and medications (use of hormone therapy, hip fracture, falls, diabetes mellitus, coronary heart disease, osteoporosis, and rheumatoid arthritis), and general health information (living alone, depression) were self-reported at baseline [18]. Height and weight of participants were measured in light indoor clothing without shoes at baseline and at 3-year follow-up, and BMI was calculated as kg/m^2^. Frequency of alcohol consumption (servings per week) was obtained by separate questionnaire. Smoking status was classified as current, former, or never. The following covariates were excluded from the analysis: hip fracture, falls, depression, diabetes mellitus (treatment by insulin, tablet or diet), coronary heart disease, and rheumatoid arteritis because they had no association with the protein intake and frailty status in bivariate correlation analysis (*P* > 0.10).

### Statistical analysis

No significant interaction by intervention (vitamin D and calcium supplementation) was observed on the association between protein intake and frailty. Hence, Data were pooled for total population (intervention and control groups). However, to account for the possible effect of vitamin D and calcium intervention on frailty and its components, analysis was adjusted for intervention group.

Protein intake g/kg BW was used as a continuous variable, per 25% increase, and as a categorized variable according to NNR 2012 (< 1.1 vs. ≥ 1.1 g/kg BW), and in quartile. In addition, protein intake g/d was also used, per 25% increase in the analyses with incident of frailty as an outcome. Sociodemographic, clinical, anthropometric, and dietary measures were compared across protein intake groups (< 1.1 vs. ≥ 1.1 g/kg BW) using Chi-square (categorical variables) and independent sample t-tests, as appropriate. Further, tests for linear trends across tertile of animal and plant protein intake (g/kg BW) were conducted using the median of each category as a continuous variable in model 2.

A series of multinomial logistic regression models were used to examine associations between protein intake and incident frailty, where the response variable was coded as not frail (reference), pre-frailty (frailty score of 1–2), or frail (frailty score of 3–5). Two models were developed with progressive adjustment for the main confounders. Model 1 was adjusted for age (years), and energy intake (kJ/d). Model 2 was adjusted for variables in model 1 plus intervention groups, height (m) to account for body size, alcohol use (servings per week), current smoking (current, former, and never), hormone therapy uses (yes or no), living alone, and income per month (euros) (surrogate for socioeconomic status). The collinearity between body weight (as confounder) and protein intake expressed as g/kg BW was strong (*P* < 0.0001) (dependent variable), thus body weight were excluded from the model [23]. Analysis of protein intake g/d, per 25% increase, was adjusted for confounders in model 2 and body mass index (kg/m^2^) was replaced with height. Models examining incident frailty with animal protein intake were adjusted for vegetable protein intake and vice versa. All statistical analyses were conducted using SPSS software version 24 for Windows (IBM Corp., Armonk, NY). All tests were two-sided and a *P* value < 0.05 was considered significant.

## Results

At the 3-year follow-up, 8.1% (*n *= 36) of women were classified as frail, and 46.8% (*n *= 206) met the criteria for prefrailty. Mean total protein intake was 0.96 g/kg BW (68.1 g/d) of which animal protein was 0.63 g/kg BW (44.8 g/d), and plant protein was 0.33 g/kg BW (21.5 g/d). The ratio of animal to plant protein intake was 2.20. Women with a protein intake ≥ 1.1 g/kg BW were less likely to be current smokers and use hormone therapy. Higher protein intake was associated with lower BMI and obesity (Table 1). When compared to those with protein intake < 1.1 g/kg BW, women consuming ≥ 1.1 g protein/kg BW had lower frequency of belonging to the lowest quartile of walking speed adjusted for height and BMI-adjusted grip strength. Protein intake g/d, per 25% increase, was equivalent to a 12.2 g/d increase in intake, and protein intake g/kg BW, per 25% increase, was equivalent to a 0.22 g/kg BW increase in intake.

Protein intake g/kg BW as continuous variable was associated with lower frailty score in the regression analyses (β coefficient = − 0.225, 95% CI = − 1.12 to − 2.30 and *P* = 0.001) and results remained significant adjusting for confounders in model 2 (results not shown). Dietary protein intake ≥ 1.1 g/kg BW was associated with a lower prevalence of prefrailty (OR = 0.45 and 95% CI = 0.01–0.73) and frailty (OR = 0.09 and CI = 0.01–0.75) at the 3-year follow-up, after adjusting for confounders in model 2.

Further, continuous protein intake as g/kg BW and g/d, per 25% increase, was assessed with risk of frailty as outcome. Protein intake g/kg BW was associated with a lower likelihood of prefrailty (OR = 0.52 and CI = 0.29–0.93) and frailty (OR = 0.13 and CI = 0.02–0.60) at the 3-year follow-up (Table 2). Also, protein intake g/d, per 25% increase, was associated with a lower likelihood of frailty (OR = 0.20 and CI = 0.32–0.99), but not prefrailty. When characterizing the exposure as absolute protein intake in quartiles for grams and g/kg BW, the association was attenuated but remained significant (results not shown).

**Table 2:** Protein intake association with frailty status

Models	ORs (95% confidence interval)		
	Normal	Prefrail	Frail
	*n* = 198	*n* = 206	*n* = 36
Protein intake ≥ 1.1 g/kg BW^a^			
Model 1	Reference	0.79 (0.51–0.98)**	0.14 (0.03–0.60)**
Model 2	Reference	0.45 (0.01–0.73)**	0.09 (0.01–0.75)**
Protein intake g/kg BW, per 25% increase			
Model 1	Reference	0.54 (0.30–0.97)**	0.18 (0.03–0.29)**
Model 2	Reference	0.52 (0.29–0.93)**	0.13 (0.02–0.60)**
Protein intake g/d, per 25% increase^b^			
Model 1	Reference	0.73 (0.41–1.30)	0.31 (0.08–1.19)*
Model 2	Reference	0.70 (0.32–1.52)	0.20 (0.032–0.99)**

Tests for a linear trend across protein intake quartiles were conducted by using the median value in each category as a continuous variable in the regression models

Odds ratios (ORs) derived from multinomial logistic regression models. Model 1 was adjusted for age (years), energy intake (kJ). Model 2 was adjusted for age (years), energy intake (kJ/d), intervention group, height (m), alcohol use (portions per week), current smoking (current smokers), hormone therapy use (yes or no), living alone, and income per month (euros)

*BW* body weight

**P value ≤ 0.05; *P value < 0.10

^a^The reference category is < 1.1 g/kg body weight

^b^Analysis of protein intake 25% increment (g/d), was adjusted for confounders in model 2 and height was replaced with body mass index (kg/m^2^)

Table 3 presents the association of a protein intake ≥ 1.1 g/kg BW with components of frailty at the 3-year follow-up. A protein intake ≥ 1.1 g/kg BW was associated with a lower prevalence of belonging to the lowest quartile of walking speed (OR = 0.53 and CI = 0.28–0.99) and belonging to the lowest quartile of grip strength adjusted for BMI (OR = 0.48 and CI = 0.26–0.89). At the 3-year follow-up, subjects in the second and third tertile of animal protein, but not plant protein, had a lower prevalence of frailty (*P* for trend = 0.019) compared to those in the first tertile; the results remained significant after adjusting for variables in model 2 (Table 4).

**Table 3:** Protein intake association with components of frailty status

	Protein intake < 1.1 vs. ≥ 1.1 g/kg body weight	
	OR (95% confidence interval)	
	Model 1	Model 2
Belong to lowest quartile of grip strength adjusted for BMI (kg/m^2^)	0.53 (0.30–0.94)*	0.48 (0.26–0.89) *
Belong to lowest quartile of walking speed adjusted for height (m/s)	0.51 (0.29–0.92)*	0.53 (0.28–0.99) *
Belong to lowest quartile of physical activity	0.72 (0.43–1.23)	0.69 (0.38–1.24)
Exhausted^a^	0.79 (0.37–1.62)	0.93 (0.42–2.03)
Weight loss > 5% of body weight	0.79 (0.36–1.73)	0.97 (0.43–2.20)

*P value < 0.05

^a^life-satisfaction score 4–12

Odds ratios (ORs) derived from logistic regression models. Protein intake < 1.1 g/kg body weight was set as the referent (*n *= 301)

Model 1 was adjusted for age (years), energy intake (kJ). Model 2 was adjusted for age (years), energy intake (kJ), intervention group, height (m), alcohol use (portions per week), current smoking (yes or no), hormone therapy use (yes or no), living alone, and income per month (euros)

**Table 4:** Animal and vegetable protein intake association with frailty status

Dietary protein intake (g protein/kg body weight)				
	Tertile 1	Tertile 2	Tertile 3	*P* for trend
Animal protein				
Tertile cutoffs	< 0.51 g	0.51─0.69	≥ 0.70	
*n*	146	147	147	
Frailty				
Model 1	1.00	1.28 (0.57─2.89)	0.18 (0.38─0.90)	0.037
Model 2	1.00	0.76 (0.33-0.94)	0.14 (0.28─0.72)	0.019
Prefrailty				
Model 1	1.00	0.87 (0.49─1.06)	0.76 (0.57─1.19)	0.069
Model 2	1.00	0.80 (0.46─0.98)	0.65 (0.33─1.01)	0.079
Plant protein				
Tertile cutoffs	< 0.25	0.25─0.33	≥ 0.34	
*n*	144	146	150	
Frailty				
Model 1	1.00	0.45 (0.17─1.17)	0.35 (0.10─1.42)	0.082
Model 2	1.00	0.41 (0.15─0.98)	0.29 (0.08─1.09)	0.086
Prefrailty				
Model 1	1.00	0.83 (0.49─0.96)	0.66 (0.37─0.94)	0.045
Model 2	1.00	0.58 (0.32─1.03)	0.57 (0.40─1.17)	0.080

Odds ratios (ORs) and confidence intervals (CI) derived from multinomial logistic regression models

Tests for a linear trend across tertiles of animal and plant protein intakes were conducted using the median value in each category as a continuous variable in the models

Model 1 was adjusted for age (years), energy intake (kJ/d). Model 2 was adjusted for age (years), energy intake (kJ/d), intervention group, height (m), alcohol use (portions per week), current smoking (current smokers), hormone therapy use (yes or no), living alone, and income per month (euros)

## Discussion

The main findings of this study show higher protein intake associated with a lower risk of prefrailty and frailty in older women. This finding is consistent with protein intake recommended by NNR 2012, which is the suggested amount to preserve physical function in older adults [6, 8, 24]. Although, previous studies have used different cut-off for protein intake rather than belower or ≥ 1.1 g/kg BW, in the French Three-City cohort among 1345 community dwelling subjects (aged 65 years and older), a protein intake ≥ 1.0 g/kg BW was associated with a lower prevalence of frailty (defined by the Fried criteria, and slowness indicated by low walking speed) [8]. The current study of the Kuopio OSPTPRE-Fracture Prevention provided an opportunity to examine protein intake using the latest NNR recommendations, research to date supports increasing the protein intake recommendation to a range of at least ≥ 1.1 g/kg BW to prevent frailty in older individuals; although longer-term studies are needed. The results of this study also showed that a protein intake g/kg BW and protein intake (g/d), per 25% increase, were associated with a lower likelihood of frailty. Our results are consistent with data from the Women’s Health Initiative study [7], where a 20% greater protein intake (% of energy) (the mean protein intake was 1.2 g/kg BW) was associated with a 9% lower risk of prefrailty and a 12% lower risk of frailty (defined according to Fried criteria) [7].

Several mechanisms may explain the relationship between protein intake and frailty. An attenuated response rate to anabolic stimulus of skeletal muscle could be one of the main reasons underpinning muscle mass and function loss in older adults [25]. It has been suggested that dietary protein intake (mainly essential amino acids) may increase anabolic response and prevent or slow the loss of muscle mass and decline of physical function in older people, which contributes to frailty [26]. We have previously shown that dietary protein intake was positively associated with walking speed and grip strength in this population [22], which are both valid indicators of disability and frailty in older adults [27, 28]. A randomized control trial conducted by Tieland et al. assessed the impact of 24 weeks of dietary protein supplementation on muscle mass, muscle strength, and physical performance in frail elderly people (*n* = 65) [29]. Subjects received either daily protein or placebo supplementation (15 g protein at breakfast and lunch); study results showed improved physical performance with dietary protein supplementation, but it did not increase skeletal muscle mass in frail elderly people. However, evidence is not consistent about the effects of protein supplementation in healthy elderly individuals [30]. While longer-term protein supplementation trials are still pending, observational studies such as this can complement findings of controlled clinical trials on higher protein intake and frailty.

Our results showed that women in the higher tertiles of animal protein, but not plant protein intakes, had a lower likelihood of frailty. However, in the Rahi et al. study, the associations between animal and vegetable protein sources and frailty were not significant [8]. Also, in the Women’s Health Initiative data, associations of total protein intake with the incidence of frailty were independent of the source (animal and vegetable). Thus, results regarding association between sources of protein intake and frailty are inconclusive and further studies are required.

In this study, due to the relatively young cohort (65–72 years old), the prevalence of frailty (8.1%) was relatively low: however, prefrailty (46.8%) was highly prevalent and it was associated with several adverse health outcomes such as higher BMI (data not shown). A unified definition for frailty is still a matter of debate; however, the proportions of frailty and prefrailty in our study were similar to previous studies [2, 5, 8]. In the Cardiovascular Health Study, prevalence of frailty among women aged 65–70 years was 3.0% [2]; in the study by Rahi et al. [8], in community-dwelling older adults aged 65 and over (*n* = 1345), prevalence was 4.1%; and in the Women’s Health Initiative study among 24,417 eligible women aged 65–79, frailty prevalence was 13.5%. It is noteworthy that our study found lower odds ratios for protein intake associated with prefrailty and frailty when compared to previous studies [7, 8]. Factors such as small sample size, and using surrogate, but not identical criteria to define frailty as suggested by Fried et al. [2] may explain this difference.

Muscle protein metabolism is greatly dependent upon ingesting an adequate amount of proteins and amino acids, which largely increases muscle protein synthesis rates and inhibits protein breakdown [31]. Thus, increase in the protein consumption to amount suggested by NNR ≥ 1.1 g protein/kg BW can be beneficial to maintain muscle mass and physical capacity in older adults. Although the exact cellular mechanism is under investigation, some studies suggest that basal rates of muscle protein synthesis may be declined somewhat with age due to suppressed stimulation of muscle protein synthesis with dietary amino acid ingestion [32]. A higher protein consumption has been suggested as an effective approach to impede “anabolic resistance” which occurs in older adults [32].

There are some limitations that should be considered in the interpretation of our findings. A range of potential confounders may have affected frailty status, and although we adjusted for a wide range of potential confounders, the possibility of other residual confounding factors cannot be excluded. This was a cross-sectional study; therefore, reverse causality as frail or prefrail women decreasing their baseline protein intake during the follow-up period cannot be excluded. Using surrogates of frailty components, such as a life-satisfaction scale for exhaustion, may cause over- or underestimation of the number of frail subjects. The independent effect of physical activity and weight change on frailty cannot be controlled, because measures of assessed weight loss and physical activity are components of the frailty definition. Our data included a rather homogenous sample of Finnish older women living in the same geographical area. Also, the NNR has been mainly developed to guide dietary intake in the Nordic population. Thus, caution should be taken in the generalization of the results to the entire older population. Due to the single time point of dietary record at the baseline visit, we could not capture the long-term dietary exposure or possible changes in dietary habits.

In conclusion, this study suggests that protein intake greater or equal to 1.1 g/kg BW is associated with a lower likelihood of prefrailty and frailty in older women. We also observed a stronger association of animal protein intake (mainly from meat, poultry, fish, and egg) with frailty than that of plant protein intake. It seems that a higher protein intake with attention given to protein quality from animal protein sources may be an effective approach to promote healthy aging and prevent frailty.
